# The allogeneic umbilical cord mesenchymal stem cells regulate the function of T helper 17 cells from patients with rheumatoid arthritis in an *in vitro* co-culture system

**DOI:** 10.1186/1471-2474-13-249

**Published:** 2012-12-13

**Authors:** Qin Wang, Xiaofeng Li, Jing Luo, Li Zhang, Lihui Ma, Zhiqin Lv, Lijin Xue

**Affiliations:** 1 Branch hospital in Fengxian of shanghai No. 6 people’s hospital, Shanghai Fengxian Center Hospital, Shanghai, China; 2 The Second Hospital Affiliated of Shanxi Medical University, Shanxi, China

**Keywords:** Mesenchymal stem cell, Peripheral blood mononuclear cells, Rheumatoid arthritis, T helper 17 cells

## Abstract

**Background:**

Previous *in vivo* studies have shown that mesenchymal stem cell (MSC) transplantation significantly improves the condition of a number of autoimmune diseases including autoimmune cerebrospinal meningitis, multiple sclerosis, glomerulonephritis and systemic lupus erythematosus.

**Methods:**

To investigate the immunoregulatory effect of stem cell transplantation, human umbilical cord MSCs were co-cultured with peripheral blood mononuclear cells (PBMCs) from patients with rheumatoid arthritis (RA). Orphan nuclear receptor gamma (ROR-γ) mRNA and protein expression was detected with real-time PCR and Western blotting. Interleukin (IL)-17, IL-6 and tumor necrosis factor (TNF-α) in the cell culture supernatant were measured using a flow cytometric bead capture method.

**Results:**

After 72 hours of co-culture, the mRNA and protein expression levels of ROR-γ in co-cultured PBMCs were decreased compared with that in PBMC of RA patients cultured alone (p < 0.05). Moreover, the decrement was positively related to the disease activity of RA (p < 0.05). Decreased secretion of IL-17, TNF-α and IL-6 were also found in co-culture supernatants of PBMCs from patients with severe and moderate disease activity, but not in supernatant from PBMCs cultured alone. The decreased cytokine expression levels were positively correlated to the concentrations of MSCs. In contrast, PBMCs from healthy controls or patients with mild RA did not show significant differences in ROR-γ expression or cytokine secretion following co-culture with MSCs as compared with those cultured alone.

**Conclusions:**

*In vitro* co-culture with MSCs down-regulated the inflammatory response of PBMCs from RA patients with severe disease activity, but had no significant effect on PBMCs from healthy controls or patients with mild disease activity, suggesting that the immunoregulatory role of MSCs may associate with the occurrence of inflammatory mediators.

## Background

Rheumatoid arthritis (RA) is a common autoimmune disease, which is usually treated with a combination of anti-rheumatic drugs. However, the clinical remission rate only reaches 40–57% despite serious side effects [1]. Recently, mesenchymal stem cell (MSC) transplantation has provided an alternative choice for treatment of autoimmune diseases. Animal model studies have found that MSC transplantation significantly improves the autoimmune cerebrospinal meningitis, multiple sclerosis, glomerulonephritis, systemic lupus erythematosus and other autoimmune diseases [2-5]. Although the mechanism remains largely unknown, MSCs have been shown to play a role in differentiation and proliferation of T lymphocytes [6,7]. Recent studies have found that RA patients with joint inflammation have increased helper T lymphocyte cells (Th1) and IL-17 cytokine producing T lymphocytes, which lead to a high level of IL-17 in synovial fluid [8]. Besides IL-17A and IL-17F, Th17 cells also express IL-6, TNF, IL-21 and IL-26. Results from a collagen-induced arthritis (CIA) animal model indicate that IL-17 receptor signalling is a critical inflammatory regulator [8,9]. Thus, overexpression of IL-17 may be associated with the pathogenesis of RA. Orphan nuclear receptor gamma (ROR-γ) is a key transcription factor for the differentiation of Th17 cells and regulates IL-17 expression *in vivo* and *in vitro*. ROR-γ knockout mice have shown reduction of Th17 cells and decreased incidence of autoimmune diseases [10]. Therefore, investigating the interaction between MSCs and Th17 may help to understand the immunological regulation and pathogenesis of RA. In this study, we designed an *in vitro* co-culture system to observe the immunoregulatory effect of MSCs on Th17 cells of RA patients.

## Methods

### Isolation and culture of umbilical cord MSCs

Umbilical cords were obtained from the Department of Obstetrics & Gynecology at The Second Hospital of Shanxi Medical University. The umbilical cord of related healthy donors were aspirated and was approved by the Ethics Committee of the affiliated the Shanxi Medical University. Umbilical cords from full-term newborn infants were washed with phosphate buffered saline (PBS) under sterile conditions and the blood vessels were carefully removed, leaving only the Wharton gum tissue. MSCs were separated and purified by cell adhesion [11,12]. Briefly, the tissue slices were cultured at 37°C in the MSC medium with 5% CO_2_ and 95% humidity. After 5–7 days, migrated spindle-shaped MSCs were found on the bottom of culture flask. Cells were passaged once 80% confluence was reached. The third-generation of MSCs was detached by 0.25% trypsin, followed by the addition of 10% fetal calf serum (FCS, Phannacia) 3 min later. The cells were centrifuged at 1500 rpm/min for 5 min to remove the supernatant, and resuspened in serum-free medium for cell counting. The cell concentration was adjusted to 2 × 10^7^/mL. Cells were centrifuged at 1500 rpm/min for 5 min and the supernatant was discarded. Cells were diluted with 0.5 ml PBS containing 2% FCS according to the concentrate ratio. The phenotypes of MSCs were assayed by flow cytometry (Beckman Coulter FC500, USA) using the monoclonal antibodies against CD44, CD45, CD34, CD71 and CD105 (BD Pharmingen, USA). Results were analysed using CXP analysis software.

### Patient selection and specimen collection

Eighteen RA patients were selected from The Second Hospital of Shanxi Medical University. The study was approved by Shanxi Medical University Ethics Committee, and informed consent was provided to patients according to the Declaration of Helsinki. The patients were grouped according to their disease activity as assessed by the disease active score 28 (DAS28) [13]. (Table 1) The low disease activity group contained six patients with DAS28 ≤ 3.2, the moderate group contained six patients with DAS28 >3.2 and ≤5.1, while the severe symptom group had six patients with DAS28 > 5.1. The diagnostic methods were based on the revised 1987 American College of Rheumatology (ACR) diagnostic criteria for RA. RA patients with other chronic diseases, e.g. diabetes, hypertension, coronary heart disease, thyroid disease, or viral hepatitis, were excluded from this study. Of all the patients, three were male and fifteen were females, aged 23–67 years (mean age 38.2 ± 12.2). The healthy control group had six subjects, of which two males and four females were aged 24–56 years (mean age 46.1 ± 11.6). Fifteen millilitres of heparin anti-coagulated blood was drawn from each patient at the morning after a 12-hour fasting period. The blood was diluted 1:1 with Hank’s solution, and peripheral blood mononuclear cells (PBMCs) were separated by gradient centrifugation over Ficoll.

**Table 1:** Baseline characteristics

**Groups**	**DAS28 ≤ 3.2**	**3.2 < DAS28 ≤ 5.1**	**DAS28 > 5.1**	**Normal**
	**(*n* = 6)**	**(*n* = 6)**	**(*n* = 6)**	**(*n* = 6)**
Age, mean ± SD years	38.2 ± 12.2			46.1 ± 11.6
Women, no.	15(3)			6(4)
Time duration from diagnosis to inclusion, months	28.2 ± 10.2	30.6 ± 8.2	16.2 ± 6.5	(−)
Combination therapy no.	6(1)	6(3)	6(6)	(−)
DAS_28_, ^*^ mean ± SD	2.5 ± 0.5	3.6 ± 0.6	5.9 ± 0.7	(−)

^*^ DAS_28_ = Disease Activity Score in 28 joints; See Patients and Methods for description of groups.

### Co-culture of PBMCs and MSCs

The PBMCs were resuspended in L-DMEM medium (Sigma company) and were co-cultured with MSCs in different ratios. Firstly, MSC with a cell density of 2 × 10^7^/ml was imbedded in a 6-well plate, and then the numbers of MSCs were counted when they covered around 80% of the area of the cell plate. The average value of the counted number (10^7^) was set as the standard of calculating the number of PBMC in each cell. The settings are as follows: Set I, Umbilical cord MSCs (1 × 10^7^ cells per well) and PBMCs were co-cultured at ratios of 1:1 (1 × 10^7^ cells per well), 1:10 (1 × 10^8^ cells per well) and 1:50 (5 × 10^8^ cells per well); Set II, Umbilical cord MSCs (1 × 10^7^ cells per well) and PBMCs of RA patients in low disease activity group were co-cultured at ratios of 1:1, 1:10 and 1:50 as described above; Set III, Umbilical cord MSCs (1 × 10^7^ cells per well) and PBMCs of moderately active RA patients were co-cultured at ratios of 1:1, 1:10 and 1:50 as described above; Set IV, Umbilical cord MSCs (1 × 10^7^ cells per well) and PBMCs of highly active RA patients were co-cultured at ratios of 1:1, 1:10 and 1:50 as described above. In each set, PMBCs cultured alone (1 × 10^7^ cells per well) were used as the control. Normal control means that MSCs were co-cultured with PBMCs of healthy donor. All culture sets were incubated in 6-well plates in a saturated humidity incubator at 37°C with 5% CO_2_ for 72 hours. PBMC suspensions were harvested by collecting the supernatant. The cells were collected by centrifugation and the supernatant was kept at −70°C for further analysis.

### ROR-γ mRNA expression

Total RNA was extracted from PBMCs using Trizol reagent according to the manufacturer’s instruction, and stored at −70°C. cDNA was synthesized following the procedures of the product operation manual (TaKaRa Code: DRR037S). PCR reactions were set to 20 μL volume containing 10 μL of SYBR®, premix Ex TaqTM II (2 ×) (Fermentas, USA), 7 μL ultra-pure water, 0.5 uL each of sense and anti-sense primers (250 nM, synthesized by Shanghai Biological Engineering Technology Services Limited) and 2 μL of cDNA. PCR was performed in triplicate for each sample and amplification conditions were set as 85°C for 30 sec followed by 40 cycles of 85°C for 5 sec and 56°C for 31 sec. The primer sequences are: ROR-γ sense primer 5^′^-CAG TGA GAG CCC AGA AGG AC -3^′^ and anti-sense primer 5^′^-TCA TCC CAT CCA TTT TTG GT-3^′^, the product size was 139 bp. The β-actin gene was used as the internal control: the sense primer was 5^′^- GGC ACC CAG CAC AAT GAA-3^′^, and the anti-sense primer was 5^′^-GGA AGG TGG ACA GCG AGG-3^′^, the product size was 98 bp. All primers were designed using the Primer 3 software.

### Western-blotting detection of ROR-γ protein expression

Cell pellets were lysed in 1 mL protein lysate buffer (50 mM Tris, Cl pH 6.8,15 mM NaCl, 5 mM EDTA, 0.5% NP-40, 1 mM PMSF) by repeated pipetting for 100 times using a syringe. The lysates were centrifuged at 10,000 g for 5 min at 4°C. The supernatant was mixed with an equal volume of 2 × SDS buffer (100 mM tris-Cl, 200 mM DTT, 20% glycerol, 0.2% bromophenol blue and 4% SDS) and boiled for 5 min, and then cooled using ice. The supernatant was collected by centrifugation at 10,000 g for 10 minutes at 4°C. The protein was separated by SDS-PAGE and transferred to a PVDF membrane, which was then blocked by 5% BSA at 4°C overnight. The membrane was incubated with a murine anti-human ROR-γ antibody (1:1000; Santa Cruze) and a murine anti-β-actin (1:1000) in Tris-buffered saline containing 0.05% Tween-20 at 4°C overnight, followed by washing and incubation with HRP-labeled anti-mouse Ig secondary antibody (1:10,000; Santa Cruze) for 2 h at room temperature. The membrane was developed with ECL solution and the image was analysed using Gel-Pro Analyzer 4 (Media Cybernetics, USA).

### Detection of cytokine secretion

The cytokine levels in the co-culture supernatant were measured and quantitated by multiplex immunoassays using a Cytometric Bead Array (CBA) kit according to the manufacture’s instruction (BD Biosciences, San Jose, CA). The cytometric beads were coated with antibodies against IL-6, TNF-α, IFN-γ and IL-17A used to capture cytokines. Each type of bead had a unique size and fluorescence intensity that can be distinguished by flow cytometry. Cytokines bound to beads were detected by the phycoerythrin (PE) conjugated secondary antibody, which was proportional to the amount of each bound cytokine. Briefly, 50 μL bead suspension and 50 μL of each sample were mixed and incubated at room temperature for 3 h. Followed by the addition of 1 mL washing buffer to each assay tube and centrifugation at 200 g for 5 min, the beads were resuspended with 300 μL washing buffer and analysed by a FACSCalibur flow cytometry (BD Biosciences). Cytokine expressing data was analysed using Flow Cytometric Analysis Program Array software (BD Biosciences).

### Statistical analysis

SPSS 13.0 for Windows was used for statistical analysis and data were presented as mean ± standard deviation ( $\overset{―}{x}\pm s$) or frequency. The one-way ANOVA and post-hoc LSD *t*-test was used for comparison among groups; meanwhile, post-hoc Dunnett’s T3 method was used for comparison among groups with unequal variance. A *P* < 0.05 was considered statistically significant.

## Results

### Phenotype of MSCs

**Figure 1:**
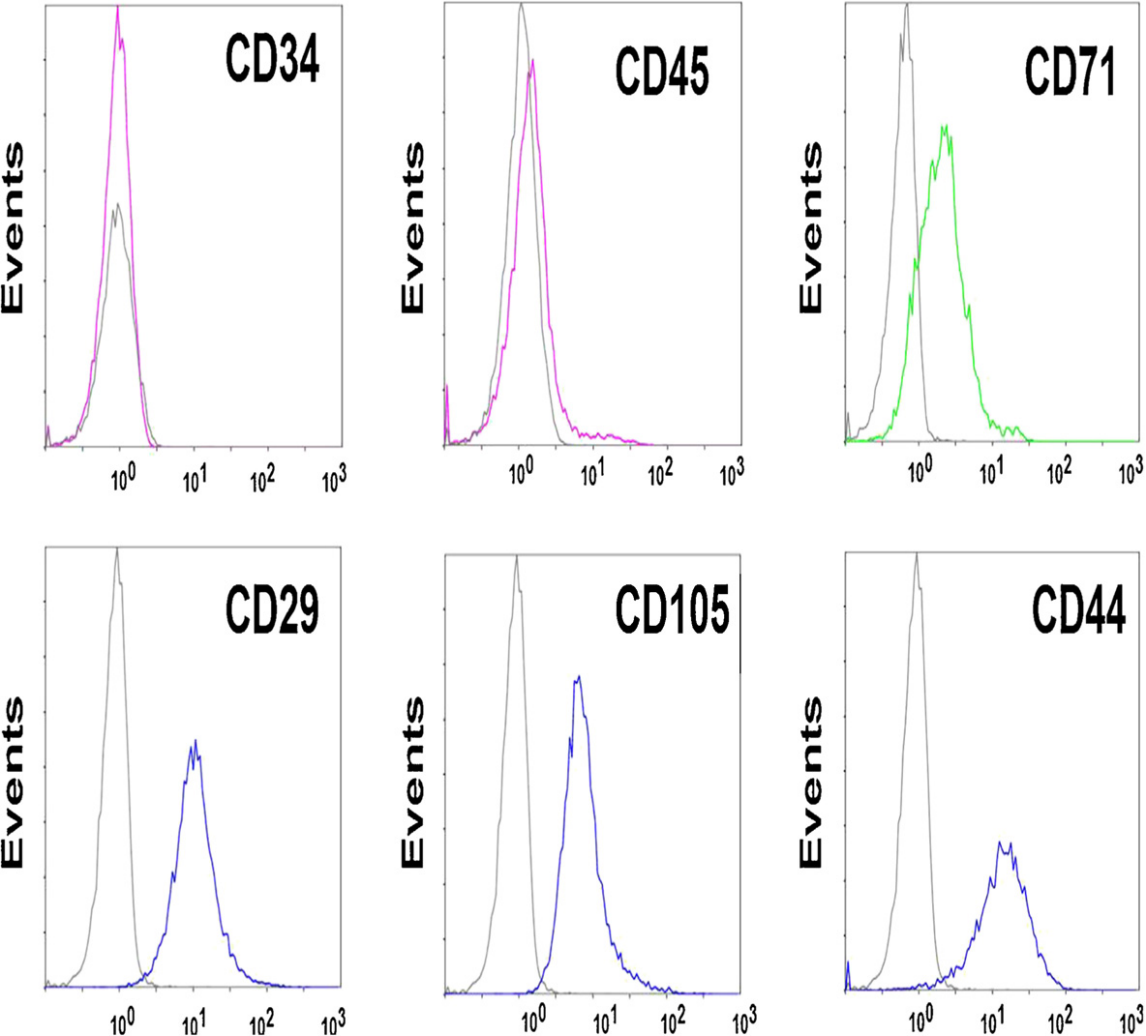
**Identification of the isolated human umbilical cord MSCs.** MSCs (3rd passage) exhibited negative for CD45 and CD34, weak positive for CD71 and positive for CD105, CD44 and CD29 as analyzed by flow cytometry.

The third passage of isolated human umbilical cord MSCs was analysed by flow cytometry. Results showed that these cells were negative for CD45 and CD34, weak positive for CD71 and positive for CD105, CD44 and CD29 (Figure 1).

### Real time PCR detection of mRNA expression for PBMC ROR-γ

**Figure 2:**
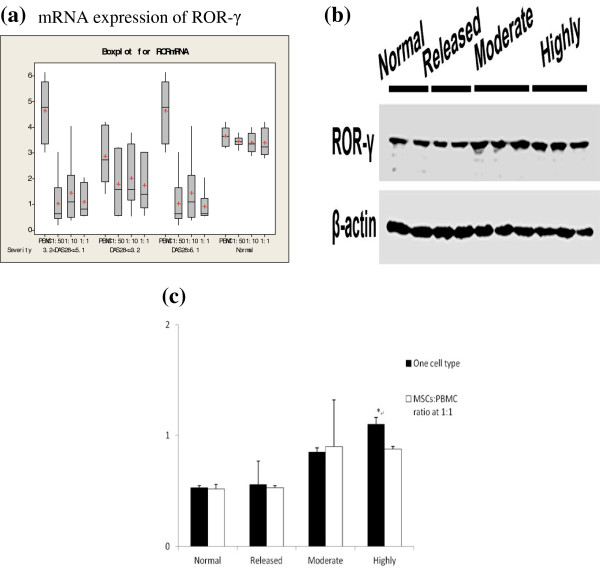
**Expression of ROR-γ. a**, mRNA expression of ROR-γ; **b**, Protein expression of ROR-γ in different groups after co-culture as measured by Western blotting. β-actin was used as control; **c**, ROR-γ expression levels in groups of MSCs: PBMC mixed ratio at 1:1 group (n = 6). *: *P* < 0.05, vs. 1:1 within the expression level.

Real time-PCR was performed to detect the mRNA expression of PBMC ROR-γ from different groups. All groups showed mRNA expression of ROR-γ. However, compared with PBMCs cultured alone, the ROR-γ mRNA expression was significantly decreased in RA PBMCs co-cultured with MSCs. No statistical difference was found in the mRNA level of ROR-γ from the low (DAS28 ≤ 3.2) and moderate (DAS28 >3.2 and ≤5) RA groups. In the severe RA group (DAS28 > 5.1), following co-culture with different ratios of co-cultured systems, there were significant differences, which was not observed in other groups. Difference was detected in the mRNA expression of PBMC ROR-γ (Figure 2a).

### Western-blotting detection of PBMC ROR-γ expression

Protein expression of ROR-γ was measured by Western blotting (Figure 2b). The results showed that the ROR-γ protein secretion levels in PBMCs of the severe RA group co-cultured with MSCs were significantly decreased as compared to that in PBMCs cultured alone. This difference was not found in any other groups (Figure 2c).

### Secretion of pro-inflammatory cytokines

**Figure 3:**
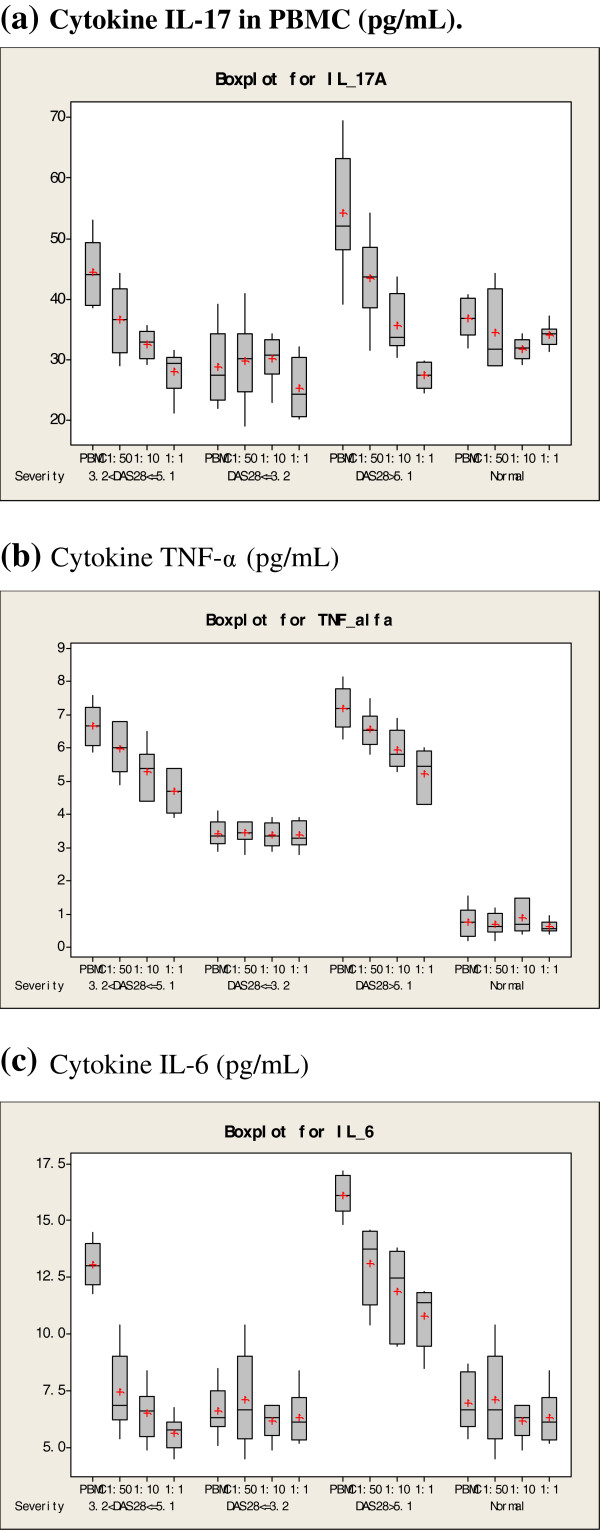
**Cytokine IL-17 in PBMC.** Cytokine TNF-α, Cytokine IL-6 expression levels in different mixed cell culture (*n* = 6). **a**, Cytokine IL-17 in PBMC (pg/mL); **b**, Cytokine TNF-α (pg/mL); **c**, Cytokine IL-6 (pg/mL).

The release of IL-17, TNF-α and IL-6 in culture supernatant was measured using CBA analysis (Figure 3a-c). The results showed that in the severe and moderate RA groups, the releases of IL-17, TNF-α and IL-6 were decreased significantly in co-cultured cells compared with other groups (p < 0.05). The decreased levels of cytokine expression were correlated to the ratio of MSCs and PBMCs, indicating that *in vitro* secretion of IL-17 by PBMCs from highly activated RA patients was inhibited by MSCs in a dose-dependent manner. In contrast, MSCs did not inhibit cytokine expression of PBMCs from mild RA patients or healthy controls.

## Discussion

This study used an *in vitro* co-culture system to investigate the interaction between MSCs and Th17 cells in PBMCs of RA patients. The biological function of Th17 cells were inhibited by co-cultured MSCs. IL-17, IL-6 and TNF-α were over-expressed in the highly active RA group, but this over-expression was suppressed to a various extent after being co-cultured with MSCs. Moreover, the inhibitory effect was correlated with the ratio of MSCs and PBMCs in the co-culture. However, the mechanisms are unclear. ROR-γ is a key transcription factor for Th17 cell differentiation and important for IL-17 expression *in vivo* and *in vitro*. Our results showed that MSCs played an inhibitory role in regulation of ROR-γ mRNA and protein expression when co-cultured with PBMC from RA patients, especially in highly active RA patients. Therefore it is possible that MSCs inhibit the function of Th17 cells via down-regulation of ROR-γ.

Interestingly, the dose-dependent inhibitory effect of MSCs was only found in high active group, MSCs in the low disease activity group and the healthy control group did not inhibit the secretion of IL-17 or other cytokines. Thus, the immune regulatory role of MSCs may depend on an inflammatory environment as activated RA patients are closely associated with high inflammatory response. On the other hand, the inhibitory effect of MSCs may be primed by a pro-inflammatory stimulus, such as lipopolysaccharide or IFN-γ. A recent study has shown that IFN-γ enhances the immune regulatory effect of MSCs, leading to promising results for the treatment of graft-versus-host disease [14]. Thus, transplantation of primed MSCs may have therapeutic potential for the treatment of RA.

## Conclusions

In this study, we found that *in vitro* co-culture with MSCs down-regulated the inflammatory response of PBMCs from RA patients with severe disease activity, suggesting that the immunoregulatory role of MSCs may associate with the occurrence of inflammatory mediators. Further study on how MSCs regulate Th17 cells in an inflammatory microenvironment may pave a road to a new therapeutic strategy for RA.

## Competing interests

The authors declare that they have no competing interests.

## Authors’ contributions

QW designed the study and performed Western blotting, participated in the whole course and drafted the manuscript. JL and LZ participated in the isolation and culture of umbilical cord MSCs, and carried out phenotypes by flow cytometry. LHM analysed mRNA expression by real-time PCR. ZQL and LJX participated in the patient selection, specimen collection and performed the statistical analysis. XFL participated in its design and helped to draft the manuscript. All authors read and approved the final manuscript.

## Pre-publication history

The pre-publication history for this paper can be accessed here:

http://www.biomedcentral.com/1471-2474/13/249/prepub

## References

[B1] Goekoop-Ruiterman YP de Vries-Bouwstra JK Allaart CF van Zeben D Kerstens PJ Hazes JM Zwinderman AH Ronday HK Han KH Westedt ML Clinical and radiographic outcomes of four different treatment strategies in patients with early rheumatoid arthritis (the BeSt study): a randomized, controlled trial Arthritis Rheum 2005 52 11 3381 3390 10.1002/art.21405 16258899

[B2] Ozaki K Sato K Oh I Meguro A Tatara R Muroi K Ozawa K Mechanisms of immunomodulation by mesenchymal stem cells Int J Hematol 2007 86 1 5 7 10.1532/IJH97.07003 17675259

[B3] Uccelli A Pistoia V Moretta L Mesenchymal stem cells: a new strategy for immunosuppression? Trends Immunol 2007 28 5 219 226 10.1016/j.it.2007.03.001 17400510

[B4] McTaggart SJ Atkinson K Mesenchymal stem cells: immunobiology and therapeutic potential in kidney disease Nephrol (Carlton) 2007 12 1 44 52 10.1111/j.1440-1797.2006.00753.x 17295660

[B5] Gonzalez MA Gonzalez-Rey E Rico L Buscher D Delgado M Treatment of experimental arthritis by inducing immune tolerance with human adipose-derived mesenchymal stem cells Arthritis Rheum 2009 60 4 1006 1019 10.1002/art.24405 19333946

[B6] Beyth S Borovsky Z Mevorach D Liebergall M Gazit Z Aslan H Galun E Rachmilewitz J Human mesenchymal stem cells alter antigen-presenting cell maturation and induce T-cell unresponsiveness Blood 2005 105 5 2214 2219 10.1182/blood-2004-07-2921 15514012

[B7] Aggarwal S Pittenger MF Human mesenchymal stem cells modulate allogeneic immune cell responses Blood 2005 105 4 1815 1822 10.1182/blood-2004-04-1559 15494428

[B8] Evans HG Gullick NJ Kelly S Pitzalis C Lord GM Kirkham BW Taams LS *In vivo* activated monocytes from the site of inflammation in humans specifically promote Th17 responses Proc Natl Acad Sci U S A 2009 106 15 6232 6237 10.1073/pnas.0808144106 19325128 PMC2669354

[B9] Cornelissen F van Hamburg JP Lubberts E The IL-12/IL-23 axis and its role in Th17 cell development, pathology and plasticity in arthritis Curr Opin Investig Drugs 2009 10 5 452 462 19431078

[B10] Ivanov II McKenzie BS Zhou L Tadokoro CE Lepelley A Lafaille JJ Cua DJ Littman DR The orphan nuclear receptor RORgammat directs the differentiation program of proinflammatory IL-17+ T helper cells Cell 2006 126 6 1121 1133 10.1016/j.cell.2006.07.035 16990136

[B11] Petsa A Gargani S Felesakis A Grigoriadis N Grigoriadis I Effectiveness of protocol for the isolation of Wharton’s Jelly stem cells in large-scale applications In Vitro Cell Dev Biol Anim 2009 45 10 573 576 10.1007/s11626-009-9227-0 19609625

[B12] Wang HS Hung SC Peng ST Huang CC Wei HM Guo YJ Fu YS Lai MC Chen CC Mesenchymal stem cells in the Wharton’s jelly of the human umbilical cord Stem Cells 2004 22 7 1330 1337 10.1634/stemcells.2004-0013 15579650

[B13] Federico NS Dolores RM Blanca H Victoria NC Sara M Mireia B Eva PP Juan GR DAS-28-based EULAR response and HAQ improvement in rheumatoid arthritis patients switching between TNF antagonists BMC Musculoskelet Disord 2009 10 1 91 10.1186/1471-2474-10-91 19627609 PMC2724400

[B14] Polchert D Sobinsky J Douglas G Kidd M Moadsiri A Reina E Genrich K Mehrotra S Setty S Smith B IFN-gamma activation of mesenchymal stem cells for treatment and prevention of graft versus host disease Eur J Immunol 2008 38 6 1745 1755 10.1002/eji.200738129 18493986 PMC3021120

